# Fatigue Life Prediction of 25CrMo4 Alloy Steel Based on Interpretable Methods

**DOI:** 10.3390/ma19122544

**Published:** 2026-06-12

**Authors:** Ze-Cheng Li, Xiao-Min Chen

**Affiliations:** 1 International Institute of Engineering, Changsha University of Science and Technology, Changsha 410114, China; 202327050303@csust.edu.cn; 2 College of Mechanical and Vehicle Engineering, Changsha University of Science and Technology, Changsha 410114, China

**Keywords:** 25CrMo4, fatigue life prediction, machine learning, SHAP method

## Abstract

The fatigue failure of railway axles is directly associated with the operational safety of trains. As 25CrMo4 steel is commonly employed for high-speed train axles, precise evaluation of its fatigue life is essential for transportation reliability. This study compared six machine learning models following hyperparameter optimization via a differential evolution algorithm. The DE-optimized Gaussian process regression (DE-GPR) model exhibited superior predictive performance, achieving a coefficient of determination (*R*^2^) of 0.8020 and a root mean square error (*RMSE*) of 0.1250 on the most significant outer test fold. Furthermore, an interpretable analysis of the model utilized a combination of SHapley Additive exPlanations (SHAP) and partial dependence plots (PDP) to elucidate feature importance. The results indicate that the applied stress level is the predominant feature affecting fatigue life predictions and that it slightly interacts with surface residual stress and full width at half maximum to influence the predicted fatigue life. This study can provide valuable insights into the fatigue life assessment and process optimization of 25CrMo4 steel components.

## 1. Introduction

Axles, as critical components of high-speed trains, bear complex cyclic loading. 25CrMo4 alloy steel, also designated as EA4T steel, has been extensively employed in the fabrication of high-speed railway axles due to its superior mechanical properties [1,2]. Various defects inevitably occur in axles during manufacturing and in-service operation, which increases the likelihood of fatigue crack initiation and poses serious potential safety hazards to train operations [3,4,5]. To extend the fatigue life of axles, multiple fatigue performance enhancement techniques have been applied, including ultrasonic surface rolling [6], induction hardening [7], and deep rolling processes [8]. Among these, shot peening, a well-established surface modification technology, can significantly improve fatigue performance by introducing compressive residual stress into the material [9]. A previous study confirmed that micro-shot peening (MSP) can simultaneously achieve maximum surface compressive residual stresses (up to −530 MPa), minimize surface roughness, and increase surface microhardness, substantially improving the material’s fatigue limit [10]. Thus, the efficient prediction of fatigue life in 25CrMo4 steel, taking into account material defects and MSP treatment, is a critical scientific issue.

Currently, the development of physics-based theoretical models is a common approach. Rooted in well-understood physical mechanisms, this method uses experimental fatigue data to validate and refine the model, thereby enhancing predictive accuracy. For example, in the Chaboche fatigue damage model, Ling et al. [11] considered the effects exerted by defects and nonlinear mean stress effects arising from the laser powder bed fusion (LPBF) process. Through parameter calibration based on experimental S-N fatigue data and defect characteristics, they successfully predicted the high-cycle fatigue life of Ti-6Al-4V alloy. However, physical theoretical models rely on empirical assumptions and mechanistic simplifications. These models may overlook the actual service conditions of railway axles. When developing a high-cycle fatigue life prediction model for TC17 alloy based on the classical Paris law of fracture mechanics, Ding et al. [12] did not consider microcrack orientation and length in their calculations. Similarly, Xu et al. [13] noted that the Paris model produces overly conservative prediction results for press-fitted axle life due to the neglect of the stress ratio effect caused by press-fitting. Although the NASGRO model developed by their research group offers improved prediction accuracy, it ignores the load spectrum effect. Consequently, inevitable errors are caused in predictions.

Machine learning models have gained widespread attention to mitigate prediction errors that arise from the incomplete analysis associated with conventional methods. Leveraging their robust theoretical foundations, machine learning approaches can effectively extract data features and quickly establish prediction models under multifactorial mechanisms, thereby overcoming the limitations of idealization [14,15]. After evaluating three different machine learning models, Zhan et al. [16] found that the random forest (RF) model achieved superior accuracy in predicting the fatigue life of SS316L material. Bao et al. [17] utilized X-ray tomography to characterize Ti-6Al-4V specimens and incorporated the extracted location, size, and morphology of internal defects into the feature set used to train a support vector machine (SVM) model, achieving a coefficient of determination (*R*^2^) of 0.99 between predictions and experimental results. Horňas et al. [18] employed a Bayesian optimization variant known as the tree-structured Parzen estimator (TPE) to optimize the hyperparameters of a gradient boosting regression (GBR) model, thus producing a highly precise predictive model. Gan et al. [19] utilized the kernel extreme learning machine (KELM), a neural network with a single hidden layer, to predict fatigue life considering mean stress effects, with the model yielding an *R*^2^ of 0.961 and a mean squared error (*MSE*) of 0.063.

Despite being constrained by the inherent “black-box” nature of machine learning models, which limits their interpretability, the specific decision-making processes of these models remain opaque [15], and the relationships between input features and predicted outcomes are not intuitive. In recent years, SHapley Additive exPlanations (SHAP) [20], which is rooted in game theory, has been applied to analyze machine learning models interpretably. Horňas et al. [21] used various machine learning models to predict the fatigue life of Ti-6Al-4V and subsequently applied the SHAP method for interpretable analysis. Their results indicated that critical features such as maximum stress and defect size exhibited a significant negative correlation with fatigue life, whereas features including defect compactness (*d_cmpt_*) and sphericity (*d_sphr_*) positively influenced the fatigue life predictions of most models. These findings closely align with existing experimental observations. Jafari et al. [22] employed machine learning to predict the brittle fracture strength of glass and ceramic materials and utilized the SHAP method to analyze feature contributions. Their results demonstrated that the dimensionless parameter ($\sqrt{t/R}$) was the most influential factor governing fracture strength, whereas Poisson’s ratio (ν) had negligible effects on the prediction. The feature importance interpreted by SHAP aligned closely with classical brittle fracture mechanics theories. Zhang et al. [23], in their study of the fatigue behavior of high-strength bolts, employed the SHAP method to examine the interaction effects among input features. Their analysis revealed that the interaction between SA and MAXS exerted the most pronounced influence on fatigue life, suggesting that a concurrent increase in both parameters should be avoided in practical engineering applications due to their synergistic detrimental effects. Conversely, the interaction between SA and MAXF exhibited an insignificant impact on fatigue performance. Yu et al. [24] also focused on feature interactions through SHAP analysis, elucidating that stress amplitude $\sigma_{\mathrm{am}}$ exhibits strong and complex interaction mechanisms with density ρ, laser volume energy density E_V_, and yield strength (YS). Specifically, elevated values of $\rho \_\sigma_{\mathrm{am}}\_\mathrm{sub}$ and $\mathrm{Ev}\_\sigma_{\mathrm{am}}\_\mathrm{sub}$ played critical roles in enhancing the fatigue life of laser powder bed fused Ti-6Al-4V alloy. Another study [25], focusing on the fatigue life of corroded steel wires, quantified the average contribution of each feature using the SHAP method. It clarified that the stress amplitude range (*S*) constitutes a key parameter influencing fatigue life. Concurrently, SHAP dependence plots revealed a threshold effect for *S* at 360 MPa, and the adverse effect of corrosion level *w* on fatigue life shows a pronounced nonlinear increase once it exceeds 5%. In conclusion, SHAP effectively quantifies the contribution of features to model outputs and analyzes the influence mechanisms and interaction effects among features, substantially enhancing the interpretability of machine learning models.

This study aims to develop an interpretable machine learning model for efficiently predicting the high-cycle fatigue life of 25CrMo4 axle steel. The differential evolution (DE) algorithm, used in conjunction with nested five-fold cross-validation [26], optimizes the hyperparameters of the candidate machine learning models. The optimal model, selected through comparative evaluation, is further analyzed by integrating SHAP with partial dependence plots (PDP). This research constitutes the first application of the SHAP-PDP joint interpretative framework to MSP 25CrMo4 steel, providing relevant insights from a machine learning perspective. Additionally, partial dependence plots are employed to verify the SHAP analysis, minimizing the risk of misinterpretation from a single method and enhancing the reliability of model explanations. By balancing predictive efficiency with interpretability, this framework provides a theoretical reference for the fatigue reliability design of 25CrMo4 alloy axles.

## 2. Data Source and Preprocessing

### 2.1. Data Source

The data utilized in this study were sourced from the notched fatigue performance experiments on 25CrMo4 alloy steel conducted by Li et al. [27]. A total of 89 rotating bending fatigue tests were performed on both MSP and untreated specimens. The dataset includes the applied stress level ($\sigma_{a}$), surface residual stress ($\sigma_{rs}$), full width at half maximum (FWHM), average roughness ($R_{a}$), equivalent notch size ($\sqrt{area}$), and corresponding fatigue life values of the specimens. These variables describe the external load, residual stress field, work hardening, surface morphology, and notch defects, rendering the dataset particularly suitable for this study.

### 2.2. Data Analysis and Preprocessing

To investigate the correlations between features, the Pearson correlation coefficient is employed to quantify linear relationships, as defined by [28]:

(1) $$r_{xy}=\frac{\sum_{i=1}^{n}\left(x_{i}-\bar{x}\right)(y_{i}-\bar{y})}{\sqrt{\sum_{i=1}^{n}{\left(x_{i}-\bar{x}\right)}^{2}}\sqrt{\sum_{i=1}^{n}{\left(y_{i}-\bar{y}\right)}^{2}}}$$

where $r_{xy}$ denotes the Pearson correlation coefficient, n represents the sample size, $x_{i}$ and $y_{i}$ are the two variables (i = 1, …, n), $\bar{x}$ and $\bar{y}$ are their averages.

**Figure 1:**
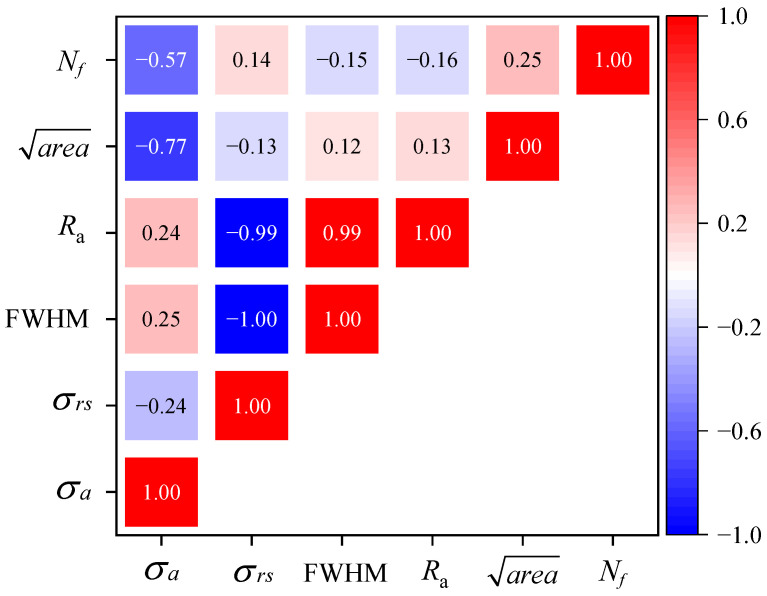
Correlation heatmap based on Pearson correlation coefficient.

As illustrated in Figure 1, a strong negative correlation exists between $\sigma_{a}$ and $\sqrt{area}$, with the most pronounced negative correlations observed between $\sigma_{rs}$ and FWHM (*r* = −1.00), and between $\sigma_{rs}$ and $R_{a}$ (*r* = −0.99). Additionally, an exceptionally strong positive correlation is detected between FWHM and $R_{a}$, with a correlation coefficient of 0.99. These findings indicate that MSP can introduce compressive residual stresses into the material, simultaneously increasing work hardening and surface roughness, consistent with the experimental results [29].

Moreover, the relationships among the input features and between each input feature and fatigue life were analyzed. A negative correlation was found between $\sigma_{a}$ and $N_{f}$ (*r* = −0.57). Conversely, weak correlations are observed between $N_{f}$ and other features: the correlation coefficient for $\sigma_{rs}$ is 0.14, for $\sqrt{area}$ is 0.25, and for FWHM and $R_{a}$ are −0.15 and −0.16, respectively.

To develop the predictive model, the dataset underwent data preprocessing, which included data cleaning, feature scaling, and target variable transformation.

During feature scaling, all input features were then standardized using the Z-score method, defined as follows [14]:

(2) $$x^{\prime }=\frac{x-\mu }{\sigma }$$

Here, x represents the original data, $x^{\prime }$ is the normalized data, μ and σ denote the mean and standard deviation of the original data, respectively.

The transformation of the target variable was necessary since the fatigue life ($N_{f}$) values varied across several orders of magnitude. To facilitate model convergence, the raw values were transformed to $\log_{10}N_{f}$.

Given the limited sample size, a fixed train–test partition was not adopted to avoid evaluation bias from a single split. Instead, 5-fold cross-validation was employed to evaluate model performance.

## 3. Predictive Models and Interpretable Analysis Methods

### 3.1. Predictive Models and Optimization Algorithms

#### 3.1.1. Paris Law

Paris law is a fundamental empirical model for describing fatigue crack propagation behavior in engineering materials. It quantifies the relationship between the fatigue crack growth rate and the stress intensity factor range, and has long been a standard benchmark for fatigue life prediction. The core formula of the Paris law is expressed as follows [30]:

(3) $$\;\frac{da}{dN}=C{\left(\Delta K\right)}^{m}$$

where da/dN is the fatigue crack propagation rate and da/dN in mm/cycle, C and m are the Paris law coefficient and exponent, respectively, with their values taken from the existing literature [27].

#### 3.1.2. K-Nearest Neighbor Regression

The K-nearest neighbor (KNN) regression, a well-established machine learning algorithm, is simple to implement. The fundamental principle involves finding the k nearest training samples in the feature space for a sample to be predicted, and the prediction is made by averaging the target values of these neighboring samples. The performance of the algorithm is influenced by the choice of distance metrics and the k value. The Euclidean distance is commonly employed, and the relevant computational formulas are presented below [31]:

(4) $$d_{E}(x,y)=\sqrt{\sum_{i=1}^{n}{\left(x_{i}-y_{i}\right)}^{2}}$$

(5) $$\hat{f}(x_{q})=\frac{1}{k}\sum_{i=1}^{k}f(x_{i})$$

where x and y are feature vectors of two samples, $d_{E}(x,y)$ denotes the Euclidean distance, $\hat{f}(x_{q})$ represents the predicted value, $f(x_{i})$ is the actual value of the *ith* nearest neighbor, k is the number of selected nearest neighbors, and *n* reflects for the total number of feature dimensions.

#### 3.1.3. Random Forest Regression

Random forest regression (RFR) is an ensemble learning technique that constructs multiple decision trees. Each tree in the training process utilizes bootstrap sampling of a fixed size on the training samples and random feature selection, promoting diversity among the trees and enhancing prediction accuracy. The calculation method using the averaging approach is shown in the formulas below [32]:

(6) $$s_{m}=\{(x_{1},y_{1}),\cdots ,(x_{m},y_{m})\}$$

(7) $${\hat{y}}_{rf}=\frac{1}{k}\sum_{i=1}^{k}f(x,s_{m}^{k})$$

where $s_{m}$ represents the data subset obtained through proportional sampling, $x_{m}$ and $y_{m}$ are the input and output vectors, respectively, ${\hat{y}}_{rf}$ is the predicted value of the target variable, x denotes the input sample, k is the number of decision trees, and $f(x,s_{m}^{k})$ indicates for the output function of the decision tree.

#### 3.1.4. Adaptive Boosting Regression

Adaptive boosting regression (ABR) is an iterative ensemble algorithm that trains a series of weak learners and adjusts the sample weights based on the prediction results from the previous iteration. The final prediction result encompasses the weighted sum of the predicted values from all weak learners. This weighted combination is expressed as follows [33]:

(8) $$H(x)=\nu \sum_{k=1}^{N}\left(\ln\frac{1}{\alpha_{k}}\right)g(x)$$

(9) $$\alpha_{k}=\frac{e_{k}}{1-e_{k}}$$

(10) $$e_{k}=\sum_{i=1}^{m}e_{ki}$$

In these equations, H(x) represents the prediction of the model for the given input sample x, ν is the regularization factor, $\alpha_{k}$ denotes the weight assigned to the *kth* weak learner, N is the total number of weak learners, g(x) signifies the median of the weighted outputs from the weak learners, $e_{k}$ indicates the total error, $e_{ki}$ represents the relative error for an individual sample, and m is the total number of samples.

#### 3.1.5. Gradient Boosting Regression

Gradient boosting regression (GBR) utilizes an ensemble model that employs decision trees as base learners. This model trains new weak learners based on the residuals from the prior weak learner to gradually reduce the overall loss [34]. By iteratively superposing multiple learners, the model can effectively approximate complex nonlinear functions with considerable accuracy. The described process involves [35]:

(11) $$F_{0}(x)=\underset{c}{\arg\min}\sum_{i=1}^{N}L(y_{i},c)$$

(12) $$c_{m,j}=\underset{c}{\arg\min}\sum_{x_{i}\in R_{m,j}}L(y_{i},F_{m-1}(x_{i})+c)$$

(13) $$F_{M}(x)=F_{0}(x)+\sum_{m=1}^{M}\sum_{j=1}^{J}c_{m,j}\cdot I(x\in R_{m,j})$$

Here, $F_{0}(x)$ is the initial model, c stands for the output parameter of the initial leaf node, $L(y_{i},c)$ defines the loss function, N is the total number of samples, $c_{m,j}$ indicates the optimal correction value for the *jth* leaf node of the *mth* regression tree, $R_{m,j}$ represents the sample region corresponding to the *jth* leaf node of the *mth* regression tree, $F_{M}(x)$ is the final prediction model, M signifies the total number of regression trees, and J is the total number of leaf nodes in a single regression tree.

#### 3.1.6. Extreme Gradient Boosting Regression

Extreme Gradient Boosting (XGBoost) is an enhanced gradient boosting regression model that uses decision trees as base learners. It iteratively trains new trees to correct prediction residuals while introducing regularization terms to control model complexity, effectively improving generalization on regression tasks [36]. The core modeling process is expressed as follows [37]:

(14) $$L^{\left(k\right)}=\sum_{i=1}^{n}l\left(y_{i},{\hat{y}}_{i}^{\left(k-1\right)}+f_{k}(x_{i})\right)+\Omega (f_{k})$$

(15) $$\Omega (f_{k})=\gamma T+\frac{1}{2}\lambda \sum_{j=1}^{T}w_{j}^{2}$$

where l is a loss function, which measures the difference between the total predicted value and ${\hat{y}}_{i}^{\left(k-1\right)}+f_{k}(x_{i})$ the target value $y_{i}$. ${\hat{y}}_{i}^{\left(k-1\right)}$ denotes the total calculation of the previous k−1 trees, and $f_{k}(x_{i})$ is the supplementary value of the *kth* tree to improve the prediction results of the previous k−1 trees. $\Omega (f_{k})$ is the regularization term of the *kth* tree, also called the greedy term, which is used to penalize the complexity of the model. Among them, T is the total number of leaf nodes of the *kth* tree, $w_{j}^{2}$ is the parameter value on each leaf node, and γ and λ are hyperparameters regulating the regularization term.

#### 3.1.7. Gaussian Process Regression

Gaussian Process Regression (GPR) is a nonparametric Bayesian regression method with a solid theoretical foundation, which can simultaneously estimate the predicted value and quantify the associated uncertainty. It is particularly suitable for nonlinear regression tasks with small datasets, and has been widely applied in fatigue life prediction fields [38,39]. In this study, the GPR model is constructed with a composite kernel function consisting of a constant kernel, a radial basis function kernel and a white noise kernel. The core modeling process is expressed as follows:

(16) y=f(x)+ε

(17) $$k(x_{i},x_{j})=\sigma_{f}^{2}\exp\left(-\frac{|x_{i}-xj{|}^{2}}{2l^{2}}\right)+\sigma_{n}^{2}\delta ij$$

Here, y is the observed target value, f(x) represents the latent regression function, ε denotes Gaussian noise. $k(x_{i},x_{j})$ is the composite kernel function of GPR, where $\sigma_{f}^{2}$ is the signal variance, l is the length scale of the radial basis function, $\sigma_{n}^{2}$ is the noise variance, and δij is the Kronecker delta function.

#### 3.1.8. Differential Evolution Algorithm

To prevent the convergence of the hyperparameter search to suboptimal solutions and to enhance prediction performance, this study adopts the DE algorithm for global optimization of the key hyperparameters in each candidate model. The DE algorithm is a population-based stochastic optimization algorithm that derives new candidates through differential mutation operations among vectors and demonstrates superior global search capability [40]. Specifically, hyperparameters such as the number of trees and maximum depth in an RF model are encoded as high-dimensional vectors, representing individuals in the DE population. After initializing a random population consisting of *N* individuals, this algorithm systematically evolves through cycles of mutation, crossover, and selection operations. A detailed description of the process is as follows [41].

In the mutation step, the vector $V_{i}^{\left(t\right)}$ is produced using a differential strategy with key equations:

(18) $$\mathrm{DE}/\mathrm{rand}/1:\;\;\;V_{i}^{\left(t\right)}=X_{r_{1}}^{\left(t\right)}+F\cdot \left(X_{r_{2}}^{\left(t\right)}-X_{r_{3}}^{\left(t\right)}\right)$$

(19) $$\mathrm{DE}/\mathrm{rand}/2:\;\;\;V_{i}^{\left(t\right)}=X_{r_{1}}^{\left(t\right)}+F\cdot \left(X_{r_{2}}^{\left(t\right)}-X_{r_{3}}^{\left(t\right)}\right)+F\cdot \left(X_{r_{4}}^{\left(t\right)}-X_{r_{5}}^{\left(t\right)}\right)$$

(20) $$\mathrm{DE}/\mathrm{best}/1:\;\;\;V_{i}^{\left(t\right)}=X_{best}^{\left(t\right)}+F\cdot \left(X_{r_{1}}^{\left(t\right)}-X_{r_{2}}^{\left(t\right)}\right)$$

(21) $$\mathrm{DE}/\mathrm{best}/2:\;\;\;V_{i}^{\left(t\right)}=X_{best}^{\left(t\right)}+F\cdot \left(X_{r_{1}}^{\left(t\right)}-X_{r_{2}}^{\left(t\right)}\right)+F\cdot \left(X_{r_{3}}^{\left(t\right)}-X_{r_{4}}^{\left(t\right)}\right)$$

(22) $$\mathrm{DE}/\mathrm{current}\text{-}\mathrm{to}\text{-}\mathrm{best}/1:\;\;\;V_{i}^{\left(t\right)}=X_{i}^{\left(t\right)}+F\cdot \left(X_{best}^{\left(t\right)}-X_{i}^{\left(t\right)}\right)+F\cdot \left(X_{r_{1}}^{\left(t\right)}-X_{r_{2}}^{\left(t\right)}\right)$$

Here, $X_{r_{1}}^{\left(t\right)}$, $X_{r_{2}}^{\left(t\right)}$, $X_{r_{3}}^{\left(t\right)}$, $X_{r_{4}}^{\left(t\right)}$, and $X_{r_{5}}^{\left(t\right)}$ signify mutually exclusive random individuals in the population, all different from the target individual $X_{i}^{\left(t\right)}$. $X_{best}^{\left(t\right)}$ represents the individual with the optimal fitness in the current generation, and the parameter F, known as the scaling factor, is a randomly generated value between 0 and 1.

During the crossover step, the trial vector $U_{i}^{\left(t\right)}$ is generated through binomial crossover:

(23) $$u_{i,j}^{\left(t\right)}=\left\{\begin{array}{ll} v_{i,j}^{\left(t\right)}, & j=j^{*}\;\mathrm{or}\;r\leq C_{r}\\ x_{i,j}^{\left(t\right)}, & \mathrm{otherwise} \end{array}\right.$$

In this equation, $j^{*}$ denotes a randomly chosen dimension, $C_{r}$ represents the crossover probability, and r is a randomly generated value in the range [0, 1].

In the selection stage, individuals are retained based on their fitness, evaluated using the following equation:

(24) $$X_{i}^{\left(t+1\right)}=\left\{\begin{array}{ll} U_{i}^{\left(t\right)}, & f(U_{i}^{\left(t\right)})\leq f(X_{i}^{\left(t\right)})\\ X_{i}^{\left(t\right)}, & \mathrm{otherwise} \end{array}\right.$$

where f(⋅) denotes the fitness function of the individual.

**Figure 2:**
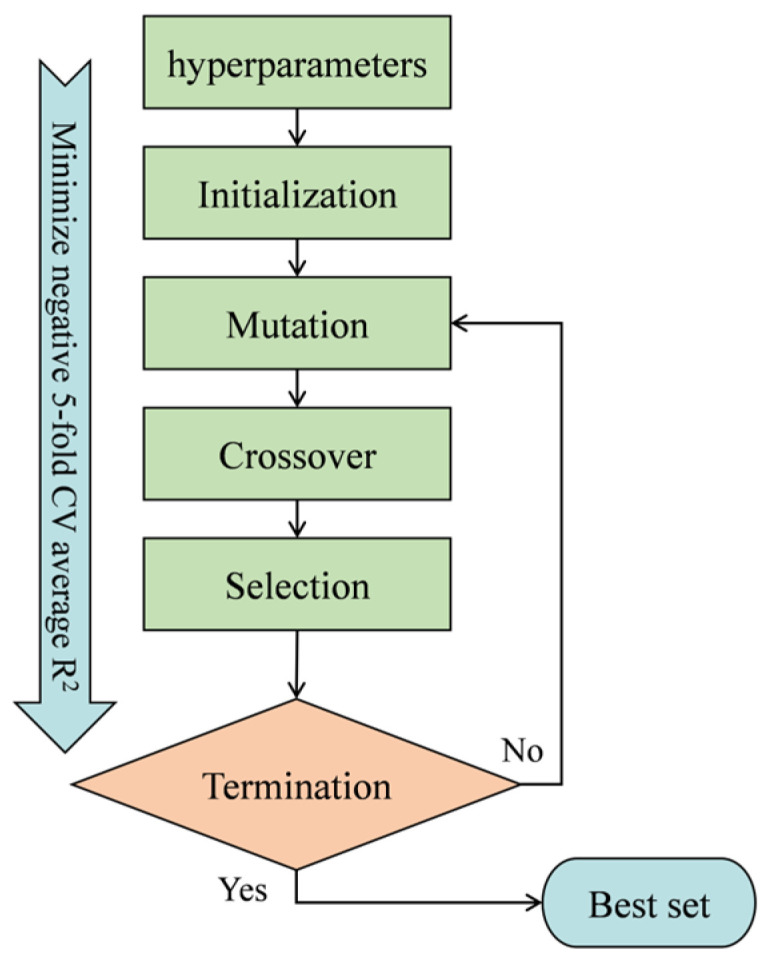
Schematic of differential evolution algorithm.

Within the nested 5-fold cross-validation framework, the fitness function for each candidate in the DE algorithm is defined as the negative average coefficient of determination (*R*^2^) calculated via 5-fold inner cross-validation on the training partition of the outer fold: $f(\cdot )=-{\bar{R^{2}}}_{\mathrm{inner}\;5\text{-}\mathrm{fold}\;\mathrm{CV}}$. This fitness metric guides the evolution of model hyperparameters toward configurations that enhance prediction performance on inner validation folds. Following iterations up to the predetermined maximum number of generations, the optimal hyperparameter combination is determined (Figure 2).

### 3.2. Model Performance Evaluation Metrics

To compare the accuracy of predictions across different models and identify the most suitable model for subsequent interpretable analysis two evaluation metrics, *R*^2^ and *RMSE*, are utilized. The *R*^2^ reflects the linear fitting degree between the predicted values and the actual values. Conversely, the *RMSE* represents the square root of the sum of squared residuals between predicted and true values, thereby quantifying the overall magnitude of the prediction errors. Both metrics were computed using the transformed fatigue life values ($\log_{10}N_{f}$), as introduced in Section 2.2, to align with the model training objectives and ensure consistent error interpretation.

During the model training and optimization phase, the average *R*^2^ derived from 5-fold inner cross-validation on the outer training partition was utilized as the primary criterion for evaluation. During the final testing and comparison phase, both *R*^2^ and *RMSE* were calculated on the independent test partitions of each outer fold. The average values across all five outer folds served as the definitive criteria for evaluating model performance. The formulas for these metrics are provided as follows:

(25) $$R^{2}=1-\frac{\sum_{i=1}^{n}{\left(y_{i}-{\hat{y}}_{i}\right)}^{2}}{\sum_{i=1}^{n}{\left(y_{i}-\bar{y}\right)}^{2}}$$

(26) $$RMSE=\sqrt{\frac{1}{n}\sum_{i=1}^{n}{\left(y_{i}-{\hat{y}}_{i}\right)}^{2}}$$

where $y_{i}$ is the true value of the sample, ${\hat{y}}_{i}$ is the predicted value generated by the model, $\bar{y}$ denotes the arithmetic mean of all true samples, and n is the total number of samples.

### 3.3. Interpretable Analysis Methods

This study establishes an interpretable analysis framework to mitigate the prevalent “black-box” challenge associated with many machine learning models. This framework incorporates SHAP and PDP.

SHAP is founded on the Shapley value concept from cooperative game theory, which allocates the output of the model prediction as a linear combination of impacts from each individual feature. The formula is presented as follows [42]:

(27) $$g(x^{\prime })=\phi_{0}+\sum_{i=1}^{M}\phi_{i}x_{i}^{\prime }$$

In this equation, g represents the explanatory model, $x^{\prime }\in {\left\{0,1\right\}}^{M}$ indicates the presence state of corresponding features, $\phi_{0}$ is the baseline constant of the model, M is the total number of input features, and $\phi_{i}$ signifies the contribution value of the feature.

Using the entire dataset, the SHAP method first calculates the baseline (mean value of the model predictions across all samples). It then measures the deviation between the prediction for an individual instance and the baseline. Each feature is considered a participant in a cooperative game, where its contribution to this deviation is quantified through its SHAP value [43]. SHAP values collectively explain the deviation from the mean prediction to that of an individual sample’s prediction. The corresponding formula is shown as:

(28) $$y_{i}=y_{base}+\sum_{j=1}^{k}f\left(X_{ij}\right)$$

Here, $y_{i}$ denotes the predicted value for the *ith* sample, $X_{ij}$ represents the *jth* feature variable of the *ith* sample, k is the total number of features, $y_{base}$ is the mean prediction value across all samples, and $f\left(X_{ij}\right)$ corresponds to the SHAP value of $X_{ij}$. The positive or negative sign of this value indicates the corresponding feature’s positive or negative impact on the predicted value of the sample.

From a game-theoretic perspective, the SHAP value for a given feature is defined as the weighted average of its marginal contributions across all possible feature subsets, represented by [20]:

(29) $$\varphi_{j}^{i}=\sum_{S}\frac{|S|!(k-|S|-1)!}{k!}\left[f_{x}\left(S\cup \{x^{j}\}\right)-f_{x}(S)\right]$$

where $x^{j}$ denotes the *jth* feature of the *ith* sample, $\varphi_{j}^{i}$ is the SHAP value of the *jth* feature for the *ith* sample, S represents all feature subsets excluding feature $x^{j}$, k is the total number of input features, |S| indicates the cardinality of set S, $f_{x}\left(S\cup \{x^{j}\}\right)$ and $f_{x}(S)$ are the model predictions with and without feature $x^{j}$, respectively.

The pure interaction effect between two features is calculated using the following equation [44]:

(30) $$\left\{\begin{array}{l} \varphi_{j,l}^{i}=\sum_{S\subseteq F\setminus \{j,l\}}\frac{|S|!\cdot (k-|S|-2)!}{2\cdot (k-1)!}\cdot \delta_{j,l}^{i}(S),\;j\neq l\\ \delta_{j,l}^{i}(S)=f_{x}(S\cup \{j,l\})-f_{x}(S\cup \{j\})-f_{x}(S\cup \{l\})+f_{x}(S) \end{array}\right.$$

where F is the complete set of input features, j and *l* represent two distinct features, $\delta_{j,l}^{i}(S)$ denotes the second-order marginal contribution of features j and l under feature subset S, and $\varphi_{j,l}^{i}$ is the pure interaction effect between feature j and feature l.

To validate and complement the SHAP analysis, PDP is employed as an auxiliary interpretative tool in this study. PDP quantifies the marginal effect of a single feature on model predictions by averaging over the remaining features [45], thereby demonstrating how the target variable varies as the feature changes. For a single feature, the PDP is defined as follows [46]:

(31) $${\hat{\mathrm{PD}}}_{j}(x_{j})=\frac{1}{n}\sum_{i=1}^{n}\hat{f}\left(x_{j},x_{-j}^{\left(i\right)}\right)$$

where $x_{j}$ represents the target feature of interest, n is the total number of training samples, $X_{-j}^{\left(i\right)}$ denotes the feature vector of the *ith* sample with the *jth* feature removed, and ${\hat{\mathrm{PD}}}_{j}(x_{j})$ refers to the average model prediction output when the *jth* feature of all samples is uniformly set to the fixed value $x_{j}$.

**Figure 3:**
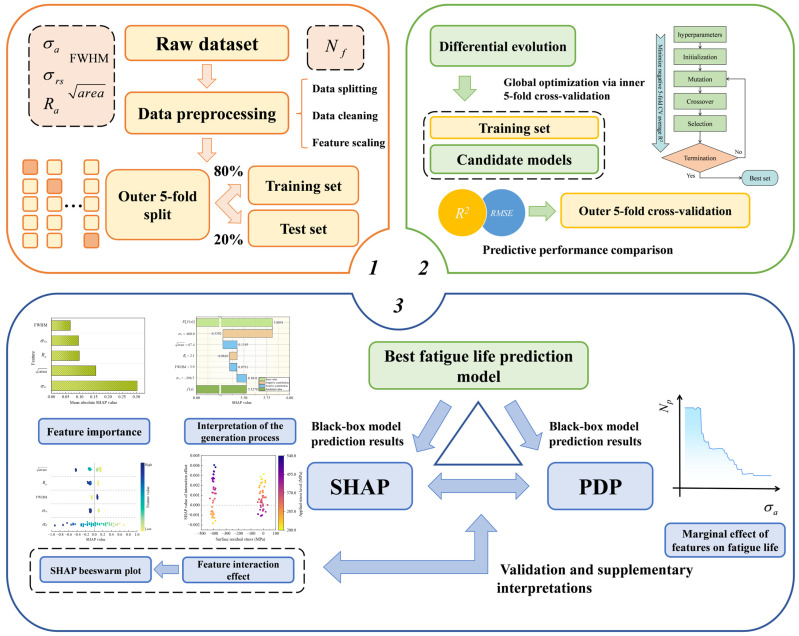
Explainable fatigue life prediction framework for 25CrMo4 steel.

In summary, this study establishes a machine learning framework for predicting the fatigue life of 25CrMo4 steel, encompassing model construction, performance evaluation, and interpretability analysis. Implemented in a Python 3.9.12 environment with scikit-learn version 1.6.1, the overall workflow consists of the following steps: First, the fatigue life dataset undergoes preprocessing; second, global hyperparameter optimization is performed for six candidate machine learning models using the DE algorithm combined with nested 5-fold cross-validation. In this method, the outer 5-folds evaluate generalization performance, while the inner 5-folds guide the DE search; third, the model demonstrating the best average performance across the outer folds is selected, and its optimal fold is extracted for subsequent interpretable analysis. The combined SHAP-PDP approach is employed to assess feature importance, analyze the influence mechanisms of individual features, dissect the prediction process of representative samples, and elucidate interaction patterns between features. The complete workflow is illustrated in Figure 3.

## 4. Results and Discussion

### 4.1. Model Prediction Results

The machine learning models described in Section 3.1 were utilized to predict the fatigue life of 25CrMo4 steel. Global hyperparameter optimization was conducted using a nested five-fold cross-validation paired with the DE algorithm. The final performance metrics, average *R*^2^ and *RMSE* along with their standard deviations, obtained from the outer five-fold cross-validation, were employed to select the optimal prediction model.

The performance metrics for each model from the outer five-fold cross-validation are presented in Table 1. The results reveal that the GPR model achieved the best overall prediction performance, registering an average R^2^ of 0.6630 ± 0.1243 and an average *RMSE* of 0.1705 ± 0.0375, surpassing the other five machine learning models. Consequently, the DE-optimized Gaussian Process Regression (DE-GPR) was preliminarily chosen as the foundational model for further interpretability analysis.

**Figure 4:**
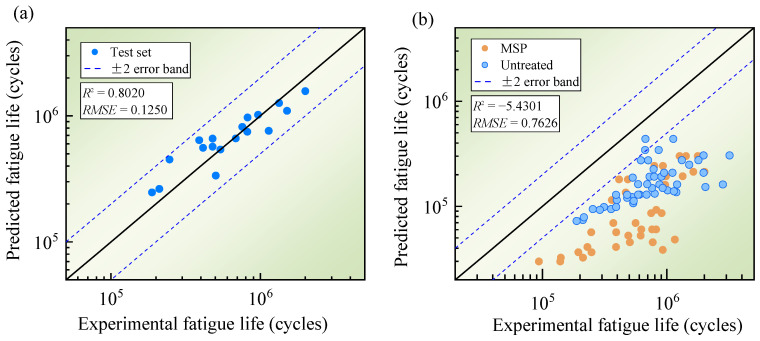
Prediction scatter plots of fatigue life: (**a**) DE-GPR model; (**b**) Paris law.

To validate further the predictive accuracy of the DE-GPR model and to juxtapose it with traditional fatigue life prediction methods, this study compared the test set prediction results from the best-performing fold of the DE-GPR model, detailing the optimal hyperparameter combination in Table 2, against the predictions derived from the classical Paris law, as depicted in Figure 4. The predicted values from the DE-GPR model for all test samples reside within the ±2× error band. In contrast, the classical Paris law typically yields conservative predictions for the fatigue life of both MSP-treated and untreated 25CrMo4 steel specimens, with all values falling below the ideal prediction line, and the majority of samples situated outside the ±2× error band. For untreated specimens, the Paris law predictions are more consistent and closer to the ideal prediction line, whereas for the MSP-treated specimens, the predictions are more dispersed and deviate further from the ideal line. This discrepancy occurs because the classical Paris law primarily accounts for the fundamental law of crack propagation and does not effectively encapsulate the crucial impacts of surface integrity elements such as compressive residual stress and work hardening introduced by MSP on fatigue life.

Existing studies [27] have demonstrated that the NASGRO crack growth model can be adapted to provide accurate fatigue life predictions for MSP 25CrMo4 steel, with results falling within a ±2× error band. Similarly, the DE-GPR model yields predictions within this error band, indicating accuracy comparable to that of the modified NASGRO model. However, the construction of the NASGRO model necessitates a profound understanding of the physical mechanisms underlying fatigue crack propagation. It depends on parameter calibration and entails a complex calculation process. By contrast, the DE-GPR model, a data-driven approach, circumvents the need for explicit physical equations and can autonomously learn the relationships among multiple variables from data, offering a straightforward and efficient prediction process. Nevertheless, it is important to recognize that the DE-GPR model operates as a “black-box” with an opaque internal decision-making process, which does not provide as intuitive and clear an understanding of the physical causal relationships between features and fatigue life as do the Paris law and NASGRO model.

Based on the feature correlation analysis results in Section 2.2, there is an extremely strong linear correlation among $\sigma_{rs}$, FWHM, and $R_{a}$, suggesting significant information redundancy among these features. To investigate the impact of this redundancy on model performance, the study designed three sets of feature reduction experiments: each involved the sequential elimination of one pair from the three highly correlated features, retaining the other three features each time to re-conduct nested five-fold DE hyperparameter optimization and model training, and comparing the results to those of the DE-GPR model with a full feature set, as shown in Table 3.

The results indicate that the performance of the models, after feature dimensionality reduction, slightly improves compared with the all-feature model. The maximum differences in the average *R*^2^ and *RMSE* were 0.0266 and 0.0065, respectively, demonstrating a certain degree of information redundancy among $\sigma_{rs}$, FWHM, and $R_{a}$. The removal of some redundant features can reduce noise interference. Although feature dimensionality reduction enhances model performance to a certain extent, given that the model with all features retains more comprehensive physical information and offers a more thorough analysis of feature contributions and interaction mechanisms for subsequent interpretability analysis, this study ultimately opted for the full-feature input DE-GPR model for further analysis.

### 4.2. Results of Interpretability Analysis

#### 4.2.1. Feature Importance

**Figure 5:**
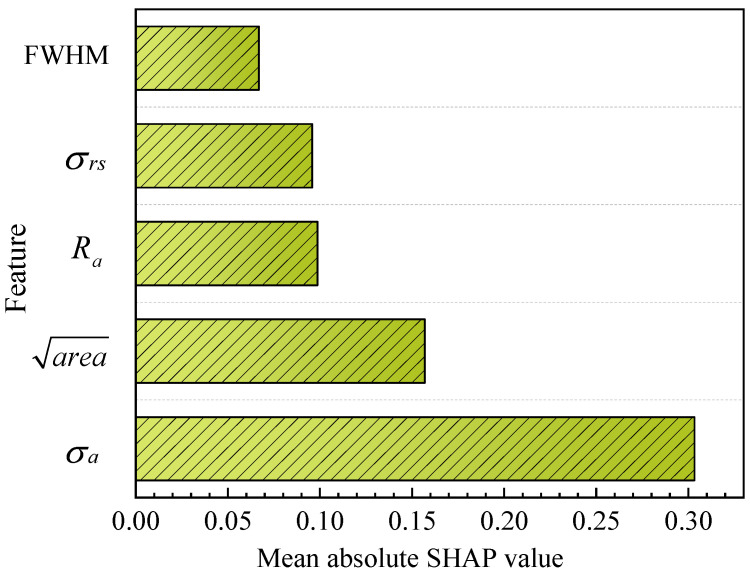
Mean absolute SHAP values for each feature.

Using the SHAP method, a global attribution analysis was initially conducted on the best fold of the outer five-fold cross-validation for the optimally configured DE-GPR model. This approach utilized the mean absolute SHAP value to evaluate the contribution of each input feature to the predicted fatigue life. As illustrated in Figure 5, $\sigma_{a}$ has the highest mean absolute SHAP value (0.3033), indicating that it is the most influential feature for the model’s predictions. The $\sqrt{area}$ follows, with a value of 0.1569, ranking second in importance. The features $R_{a}$, $\sigma_{rs}$, and FWHM have considerably lower values of 0.0987, 0.0958, and 0.0669, respectively, with FWHM contributing the least. The overall feature importance ranking for the predicted fatigue life, derived from the SHAP analysis, is as follows: $\sigma_{a}$, $\sqrt{area}$, $R_{a}$, $\sigma_{rs}$, and FWHM.

**Figure 6:**
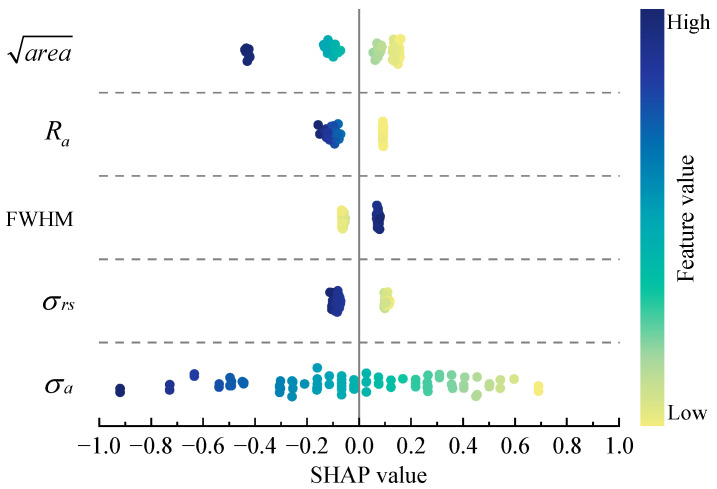
SHAP beeswarm plot for all features.

Figure 6 depicts the SHAP beeswarm plot, where each point represents the SHAP value of an individual sample, color-coded according to the magnitude of the corresponding feature. Each row demonstrates the influence of a specific feature on predicted fatigue life. High-value sample points for $\sigma_{a}$ and $\sqrt{area}$ are predominantly located on the left side of the zero axis, displaying negative SHAP values, whereas low-value samples are found on the right side with positive SHAP values. This pattern indicates their detrimental effects on the predicted fatigue life. The distribution range of $\sqrt{area}$ is narrower than that of $\sigma_{a}$, signifying its weaker overall influence on model prediction outcomes. For $\sigma_{rs}$, which ranges from −430 MPa to 34 MPa, samples exhibiting tensile residual stress show a concentrated distribution on the left of the zero axis, which negatively impacts predicted fatigue life. Conversely, samples with compressive residual stress display a concentrated distribution on the right side, contributing positively to predicted fatigue life. This pattern corroborates the findings referenced in the literature, which state that tensile residual stress is deleterious, whereas compressive residual stress is advantageous for fatigue performance [47]. $R_{a}$ exhibits a distribution trend comparable to $\sigma_{rs}$: high values correspond to negative SHAP values, and low values are associated with positive SHAP values, indicating their adverse effect on predicted fatigue life. Among all features, FWHM presents the most significant SHAP distribution differences. Elevated FWHM values correlate with positive SHAP values, reflecting the established relationship between FWHM and the square root of the dislocation density [48]; a higher dislocation density enhances plasticity and thus improves fatigue resistance.

#### 4.2.2. Interpretation of the Prediction for a Single Sample

**Figure 7:**
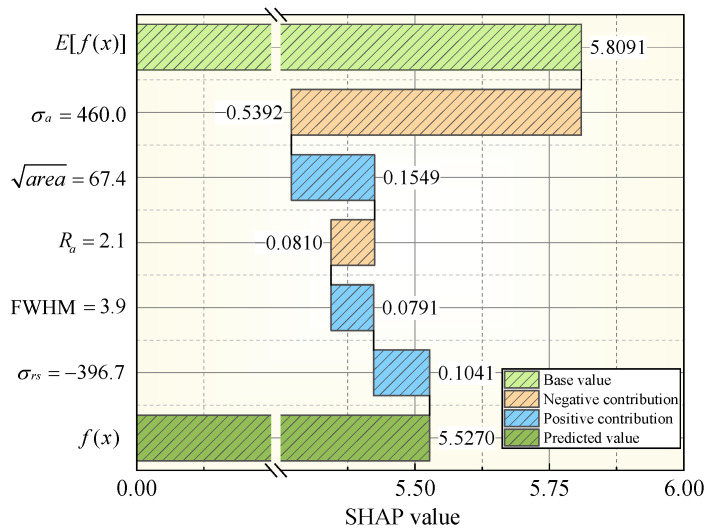
SHAP interpretation of prediction results for the 39th sample.

Figure 7 illustrates the prediction process for Sample 18 and the underlying decision logic of the model. The baseline prediction E[f(x)] derived from the SHAP method is 5.8091 (on a logarithmic scale), corresponding to the mean predicted fatigue life of the dataset, i.e.,10^5.8091^ cycles. The $\sigma_{a}$ for this sample is 460.0 MPa, significantly higher than the average in the training set. The SHAP analysis assigns a negative correction of −0.5392 to this feature, resulting in a corrected fatigue life of 10^5.2699^ cycles. This negative contribution is consistent with the established understanding that higher stress levels impair fatigue life, confirming the model’s accurate internalization of this relationship. Additionally, both the FWHM and $\sigma_{rs}$ positively influence the model prediction. Conversely, the $R_{a}$ value, which is higher than the average, provides a small negative correction of −0.0810. The actual value of $\sqrt{area}$ for this sample is 67.4, and the SHAP analysis indicates a positive contribution of 0.1549 to this feature. Consequently, the baseline prediction is adjusted to a final predicted fatigue life of 10^5.5270^ cycles. This sample clearly demonstrates the additive nature of SHAP values in decomposing model predictions.

#### 4.2.3. Feature Marginal Effects Based on PDP

**Figure 8:**
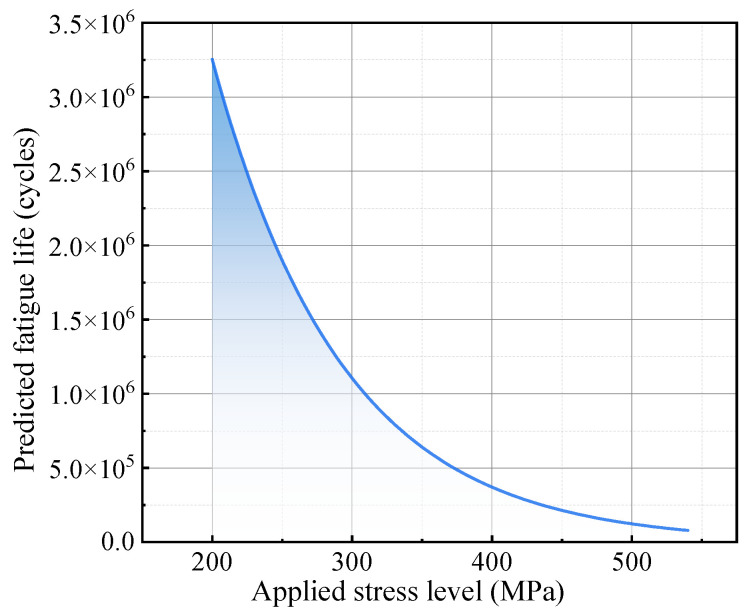
Partial dependence plot of applied stress level.

**Figure 9:**
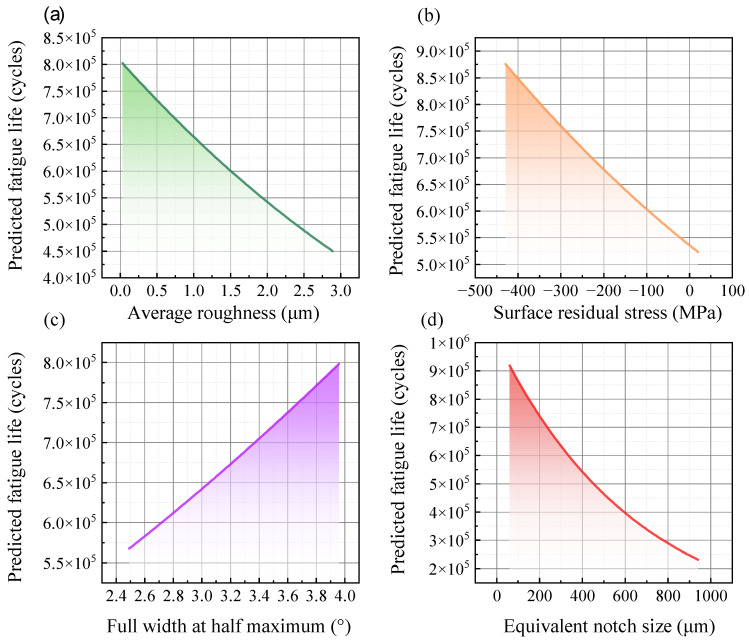
Partial dependence plots: (**a**) Average roughness; (**b**) Surface residual stress; (**c**) Full width at half maximum; (**d**) Equivalent notch size.

Figure 8 and Figure 9 display the partial dependence plots for input features, demonstrating their marginal effect on the predicted fatigue life of 25CrMo4 steel. As depicted in Figure 8, the predicted fatigue life decreases from 3.25 × 10^6^ cycles to 0.79 × 10^5^ cycles as the applied stress level increases from 200 MPa to 550 MPa. This trend is consistent with the classical S-N curve behavior, corroborating both the data reliability and the predictive capacity of the established model.

Figure 9 displays the partial dependence plots for the remaining four features. As shown in Figure 9a, the predicted fatigue life diminishes with an increase in average roughness. This observation aligns with the theory that surface roughness influences the fatigue crack growth threshold by inducing crack closure [47]. Figure 9b depicts an upward trend in predicted fatigue life as the surface residual stresses become more compressive. This pattern confirms that compressive residual stress effectively inhibits the initiation and propagation of fatigue cracks [49]. For full width at half maximum (Figure 9c), the predicted fatigue life demonstrates a gradual increase as its value rises. Conversely, as depicted in Figure 9d, the predicted fatigue life decreases with an increase in the equivalent notch size. The trends derived from the PDPs are highly consistent with those obtained in the previous SHAP beeswarm plot analysis. The consistency between these two analytical results and the established mechanisms further authenticates the model interpretation.

#### 4.2.4. Interaction Effects Among Features

**Figure 10:**
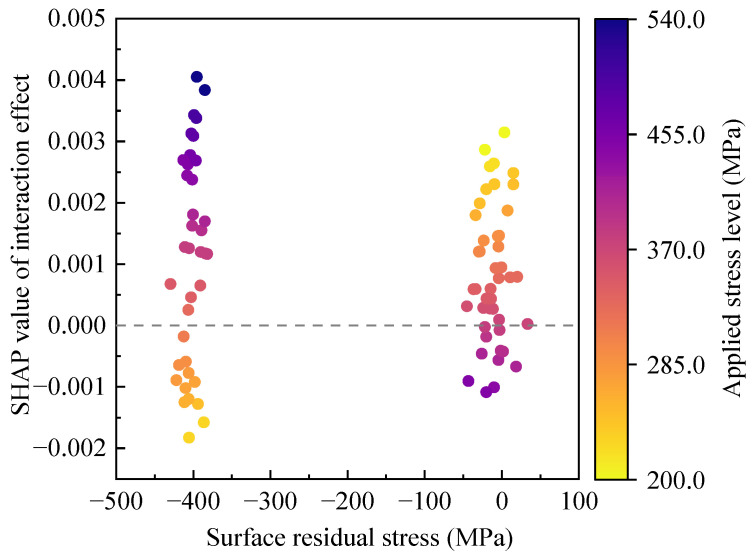
Pure SHAP interaction values between surface residual stress and applied stress level.

**Figure 11:**
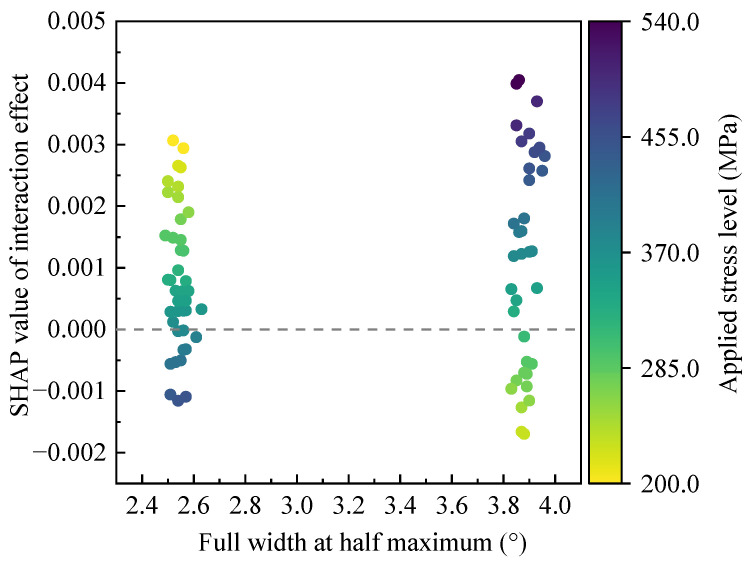
Pure SHAP interaction values between FWHM and applied stress level.

To further explore interaction effects among key features, the pure SHAP interaction values between paired features were computed. Figure 10 depicts the pure SHAP interaction values between surface residual stress and applied stress level, while Figure 11 displays those between full width at half maximum and applied stress level.

The interaction values shown in Figure 10 exhibit distinct characteristics corresponding to the two surface treatment states. In the high compressive residual stress region (approximately −400 MPa), which includes the MSP specimens, the pure interaction values range from −0.0018 to +0.0041. Higher applied stress levels tend to yield positive interaction values, while lower applied stress levels are associated with negative ones. This pattern may reflect a physical coupling mechanism between the stable compressive stress layer induced by MSP and external cyclic loading. The variation in interaction values with applied stress magnitude may be linked to residual stress relaxation [50]. For 25CrMo4 alloy steel, relaxation may be associated with the number of cycles: lower applied stress leads to longer fatigue lives, and under extended cyclic periods, relaxation is more likely to occur, potentially resulting in a weak negative pure interaction in the SHAP results.

For the low residual stress region (ranging from −50 MPa to 35 MPa), corresponding to the untreated specimens, the interaction values are distributed between −0.0010 and +0.0035. Here, higher applied stress levels correlate with negative interaction values, while relatively low applied stress levels yield slightly positive values. This distribution likely reflects the absence of an effective protective stress layer in untreated specimens. In the absence of compressive residual stress, crack propagation is primarily driven by the applied stress itself, and the interaction between the surface stress state and external loading appears relatively weak and passive.

Figure 11 elucidates two distinct interaction trends correlated with different ranges of FWHM values. For a low FWHM value (approximately 2.55), characteristic of untreated specimens, the pure interaction value exhibits a gradual shift from +0.0031 to −0.0012 as the applied stress level increases. This trend, which is monotonic, may originate from the initially low dislocation density in the untreated surface, whereby the interaction is predominantly influenced by the external applied stress. With increasing levels of applied stress, the SHAP interaction value transitions from positive to negative.

Conversely, for a high FWHM value (approximately 3.9), associated with MSP-treated specimens, the pure interaction value decreases from +0.0040 to −0.0017 as the applied stress level decreases. In this scenario, higher levels of applied stress correspond to positive interactions, whereas lower stress levels result in negative interactions. This differential trend can be attributed to the pre-established high-density dislocation network caused by MSP, which creates a work-hardened surface layer. Under conditions of high applied stress, this work-hardened layer retards crack initiation, thus positively affecting the predicted fatigue life. For 25CrMo4 alloy steel, the influence of the work-hardened layer appears to be minimal under low stress amplitudes, which could account for the predominantly negative or neutral SHAP interaction values at these lower stresses.

Although the interaction effects observed are significantly smaller than the primary effects of individual features, their orderly distribution might indicate potential synergistic relationships between surface treatment characteristics and external cyclic loading. While primary effects continue to dominate the model predictions, these secondary interaction features enrich and refine the model’s interpretation. However, the physical interpretations of SHAP interaction values, being derivations from statistical associations within the model, require further experimental validation.

## 5. Conclusions

Based on 89 sets of fatigue test data of 25CrMo4 alloy steel, six machine learning algorithms were employed to predict fatigue life. Ultimately, a DE-GPR model was established. To elucidate the model decisions, this study utilized an interpretable framework combining the SHAP method with PDP to assess the significance and influence mechanisms of each feature in predicting fatigue life, as well as to examine the interaction patterns between features. The main conclusions are summarized as follows:

1. Feature importance ranking based on mean absolute SHAP values shows that applied stress level is the most influential feature in the fatigue life predictions, followed by equivalent notch size, average roughness, surface residual stress, and FWHM. Among these, applied stress level, equivalent notch size, and average roughness exhibit a negative association with predicted fatigue life, whereas FWHM shows a positive association. The effect of surface residual stress is more complex.
2. Marginal effect analysis confirms that the variation in predicted fatigue life with applied stress level follows the typical S-N curve shape. The trends for all features are consistent with the SHAP beeswarm plot, thus enhancing the reliability of the interpretation.
3. Surface residual stress and FWHM exhibit distinct interactive characteristics with varying levels of applied stress under different surface treatment conditions. For MSP specimens within regions of high compressive residual stress, the interaction values transition from positive to negative as the applied stress level decreases. This shift may be linked to the relaxation of residual stress under long-term cyclic loading. In the case of MSP specimens with high FWHM values, the interaction values decrease gradually as applied stress declines. This observation suggests that in 25CrMo4 alloy steel, the work-hardened layer appears to contribute only minimally under low stress amplitudes.

This study successfully developed a DE-GPR model with satisfactory predictive performance for the fatigue life prediction of 25CrMo4 alloy steel. It also systematically explored the influence mechanisms of features using the SHAP-PDP interpretable framework. However, some limitations remain. The current dataset comprises only 89 samples. Although nested five-fold cross-validation was employed to rigorously evaluate the generalization performance and mitigate overfitting, the model’s applicability to broader conditions still requires confirmation with larger and more diverse datasets. Consequently, the proposed model is currently only suitable for predicting the high-cycle fatigue life of MSP 25CrMo4 steel at room temperature. Additionally, the physical mechanisms underlying the observed interaction effects necessitate further targeted experimental validation.

## Figures and Tables

**Table 1:** Comparison of average performance of six machine learning models from outer 5-fold cross-validation.

Algorithm	Average *R*^2^	Standard Deviations	Average *RMSE*	Standard Deviations
KNN	0.5244	0.2447	0.1838	0.0090
RFR	0.5173	0.1973	0.2002	0.0227
ABR	0.5623	0.2086	0.1900	0.0304
GBR	0.5274	0.1964	0.1980	0.0217
XGBoost	0.5083	0.1331	0.2057	0.0203
GPR	0.6630	0.1243	0.1705	0.0375

**Table 2:** Optimized hyperparameter combination for the DE-GPR model.

Algorithm	Parameter	Value
Gaussian Process Regression (GPR)	length_scale	36.19
	constant_value	67.25
	noise_level	0.033

**Table 3:** Performance comparison of DE-GPR models before and after feature dimensionality reduction.

Feature Combination	Average *R*^2^	Standard Deviations	Average *RMSE*	Standard Deviations
All features	0.6630	0.1243	0.1705	0.0375
Excluding $\sigma_{rs}$ and FWHM	0.6848	0.0913	0.1652	0.0276
Excluding $\sigma_{rs}$ and $R_{a}$	0.6896	0.0926	0.1640	0.0289
Excluding FWHM and $R_{a}$	0.6777	0.1014	0.1665	0.0271

## Data Availability

The raw experimental data presented in this study are available in The fatigue test and surface condition datasets of notched UP and MSP specimens at https://doi.org/10.1016/j.engfracmech.2022.108992, reference number [27]. The original contributions presented in the study are included in the article. Further inquiries can be directed to the corresponding author.
